# Early detection of diabetic neuropathy based on health belief model: a scoping review

**DOI:** 10.3389/fendo.2024.1369699

**Published:** 2024-04-24

**Authors:** Okti Sri Purwanti, Nursalam Nursalam, Moses Glorino Rumambo Pandin

**Affiliations:** ^1^ Department of Advanced Nursing, Faculty of Nursing, Universitas Airlangga, Surabaya, Indonesia; ^2^ Department of Nursing, Faculty of Health Science, Universitas Muhammadiyah Surakarta, Surakarta, Indonesia; ^3^ English Department, Faculty of Humanities, Universitas Airlangga, Surabaya, Indonesia

**Keywords:** diabetes mellitus, neuropathy, early detection of neuropathy, neuropathy instrument, neuropathy examination

## Abstract

**Introduction:**

Uncontrolled blood sugar levels may result in complications, namely diabetic neuropathy. Diabetic neuropathy is a nerve disorder that causes symptoms of numbness, foot deformity, dry skin, and thickening of the feet. The severity of diabetic neuropathy carries the risk of developing diabetic ulcers and amputation. Early detection of diabetic neuropathy can prevent the risk of diabetic ulcers. The purpose: to identify early detection of diabetic neuropathy based on the health belief model.

**Method:**

This research searched for articles in 6 databases via Scopus, Ebsco, Pubmed, Sage journal, Science Direct, and SpringerLink with the keywords “screening Neuropathy” AND “Detection Neuropathy” AND “Scoring Neuropathy” AND “Diabetic” published in 2019-2023. In this study, articles were identified based on PICO analysis. Researchers used rayyan.AI in the literature selection process and PRISMA Flow-Chart 2020 to record the article filtering process. To identify the risk of bias, researchers used the JBI checklist for diagnostic test accuracy.

**Results:**

This research identified articles through PRISMA Flow-Chart 2020, obtaining 20 articles that discussed early detection of diabetic neuropathy.

**Conclusion:**

This review reports on the importance of early detection of neuropathy for diagnosing neuropathy and determining appropriate management. Neuropathy patients who receive appropriate treatment can prevent the occurrence of diabetic ulcers. The most frequently used neuropathy instruments are the vibration perception threshold (VPT) and questionnaire Michigan Neuropathy Screening Instrument (MNSI). Health workers can combine neuropathy instruments to accurately diagnose neuropathy.

## Introduction

Diabetes mellitus may cause neuropathy, retinopathy, and nephrotic complications. The increase in the number of diabetes mellitus cases that occur if not managed properly can cause complications, some complications that occur in diabetes mellitus sufferers that occur can significantly affect the decline in the quality of life of diabetes patients so that low quality of life can affect the physical and mental well-being of diabetes patients (1). On the other hand, diabetes mellitus over a long period may be a factor that worsens the condition of heart failure patients (2). For diabetes mellitus patients, diabetic neuropathy is the most common complication in type 2 diabetes mellitus patients. Diabetic neuropathy results in decreased function of the sensory (decreased sensitivity), motor (deformity), and autonomic (callus) nerves (3). The majority of diabetics experience small wounds on the feet that lose sensitivity and develop into diabetic ulcers. Diabetic ulcers can cause infection and foot amputation (4). The health belief model estimates patient attitudes in preventing diabetic neuropathy. The health belief model includes vulnerability, benefits, obstacles, the seriousness of illness, and support received (5).

The incidence of neuropathy in the world reaches 2.4% of the human population, and the prevalence of neuropathy cases increases in old age by 8.0%. Globally, the highest prevalence of neuropathy occurs in the Asian continent. A higher incidence of neuropathy can be found in countries on the Southeast Asian continent, namely Malaysia (54.3%), the Philippines (58.0%) and Indonesia (58.0%) (6). A study showed that 50% of patients aged > 60 years experience neuropathy in the early stages of type 2 diabetes (7). Diabetic who experience complications from diabetic neuropathy in Indonesia reach 54% (8).

Early detection of neuropathy is to establish an early diagnosis of neuropathy and determine patient care. Proper treatment for neuropathy patients can prevent diabetic ulcers (9). Nurses can carry out early detection of neuropathy using neuropathy instruments before the emergence of neuropathy symptoms. Patients who are aware of the signs of neuropathy and carry out appropriate foot care can prevent diabetic ulcers (10). In fact, patients are willing to undergo a neuropathy examination if the patient feels the severity of neuropathy symptoms. Health workers make a diagnosis of neuropathy after clinical signs of neuropathy appear (11).

Based on the explanation above, early detection of neuropathy is carried out to confirm the diagnosis and prevent diabetic ulcers. This research aimed to determine early detection of diabetic neuropathy based on the health belief model.

## Methodology

**Figure 1:**
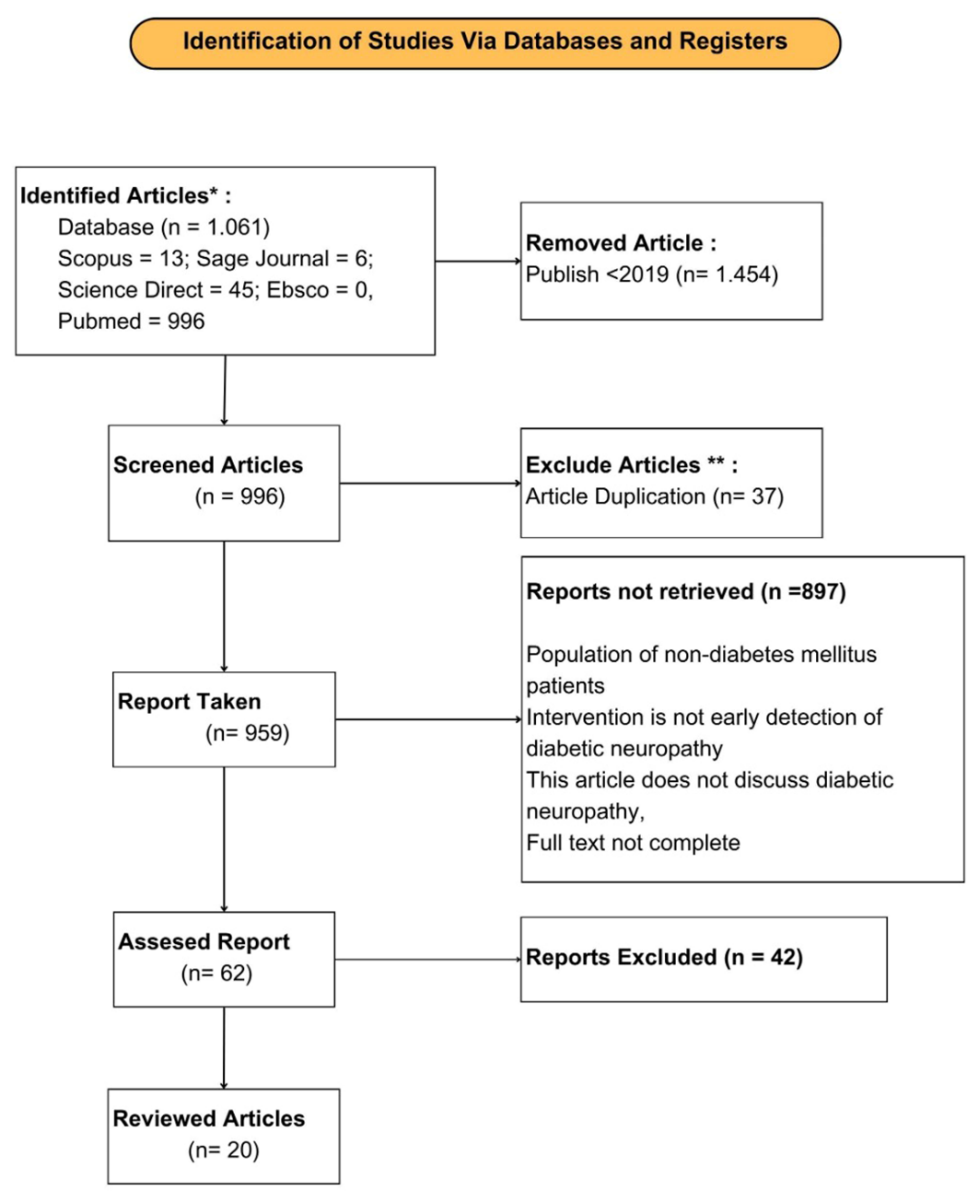
PRISMA flow chart 2020 diagram.

This research used a scoping review approach. The initial stage of this research was identifying problems based on existing phenomena. Next, the researcher determined inclusion and exclusion criteria in literature screening. The researcher compiled the final results based on the literature included in the screening process. Researchers used the PRISMA Flow chart 2020 diagram to document the literature selection process. Researchers conducted literature searches based on 6 databases, namely PubMed, Scopus, Science Direct, Sage Journal, Ebsco and SpringerLink. At the literature search stage, researchers used a combination of the keywords “Screening Neuropathy” AND “Detection Neuropathy” AND “Scoring Neuropathy” AND “Diabetic” in literature published in the last 5 years (2019-2023). Based on the results of the literature search, the researcher downloaded the articles and carried out filtering. Researchers excluded review articles, letters to the editor, subchapters from books, and articles that were incomplete. Researchers carried out literature screening analysis that was explained in the inclusion and exclusion criteria. The literature selection process used Rayyan. AI by inputting literature search results on the website. In the initial stage of literature selection, researchers remove duplicate literature that was detected. Next, select articles based on title, abstract, full text. Documentation of the literature selection process using the PRISMA Flow chart 2020 diagram in **Figure 1**. Data extraction based on the results of the literature selection, the researcher carried out data extraction including the following: 1. Author and year, 2. Study design, 3. Sample, 4. Variables, 5. Instrument, 6. Intervention, 7. Analysis, 8. Results. Researchers recorded all instruments used in early examination of diabetic neuropathy. The risk of bias assessment in this review uses a critical appraisal checklist that is available from the Joanna Brings Institute (JBI). Researchers used the JBI diagnostic test accuracy checklist to assess the risk of bias across the literature. The JBI diagnostic test accuracy checklist can be used in literature assessments with cross-sectional and case study research designs. Risk bias if an assessment of ≥50% is considered to meet critical assessment criteria (12). The risk of bias results can be seen in **Table 1**.

**Table 1:** Critical appraisal of eligible diagnostic test accuracy.

Citation	Q1	Q2	Q3	Q4	Q5	Q6	Q7	Q8	Q9	Q10	Total	Note
(13)	1	1	0	0	0	1	1	1	1	1	6	Eligible
(14)	1	1	0	0	0	1	0	1	1	1	6	Eligible
(15)	1	1	0	1	0	0	1	1	0	1	6	Eligible
(16)	0	1	1	1	1	1	1	0	0	1	7	Eligible
(17)	0	1	0	1	1	1	1	1	1	1	8	Eligible
(11)	1	1	1	0	1	1	0	0	1	1	7	Eligible
(18)	1	1	0	0	1	1	1	1	0	1	7	Eligible
(19)	1	1	0	1	1	1	1	0	1	0	7	Eligible
(20)	1	1	0	0	0	1	0	1	1	1	6	Eligible
(21)	1	1	1	1	1	0	1	0	0	1	7	Eligible
(4)	1	0	0	1	1	1	0	1	1	1	6	Eligible
(22)	1	1	1	0	0	1	0	0	1	1	6	Eligible
(23)	1	1	0	1	0	1	1	0	1	1	7	Eligible
(24)	1	0	0	1	1	1	1	1	1	1	8	Eligible
(25)	0	1	1	1	1	0	1	1	1	0	7	Eligible
(26)	1	1	0	1	0	1	0	1	0	1	6	Eligible
(27)	1	1	0	0	1	1	1	1	1	1	8	Eligible
(28)	1	1	0	1	1	1	1	0	0	1	7	Eligible
(29)	1	1	0	0	1	1	1	1	1	1	8	Eligible
(30)	0	1	0	1	0	1	1	0	1	1	6	Eligible
Percentage	60.0	70.0	15.0	60.0	60.0	85.0	65.0	55.0	65.0	75.0		

(31).

## Results

In this study, there were 1,061 pieces of literature that were included in the screening process. The researcher identified duplicate literature and removed them. Next, the literature was selected based analysis to obtain the final results of the literature to be reviewed. Based on the results of the selection of literature included in this review, there were 20 pieces of literature. The literature research design was divided into 2 types, namely 18 literatures with a cross sectional study design and 2 literatures with a case control study design. The results of the selection of literature to be reviewed can be seen in the PRISMA Flow Chart 2020 diagram at **Figure 1** (32).

Based on the final results of the literature screening, showed that early detection of neuropathy can be done using several methods that will be described as follows **Tables 2**, **3**.

**Table 2:** Journal review.

Author	Study design	Sample	Variable	Instrument	Intervention	Analysis	Results
(13)	Cross-sectional study	34 patients	Independent: Toronto clinical neuropathy score (TCNS) and modified Toronto clinical neuropathy score (m TCNS) Spanish version Dependents: Diabetic polyneuropathy	Toronto clinical neuropathy score Spanish version and modified Toronto clinical neuropathy score Spanish version	Neuropathy examination using Toronto Clinical Neuropathy Score (TCNS) Spanish version.	Cronbach’s alpha	The validity test used Cronbach’s alpha with a TCNS result of 0.83 and m TCNS of 0.85. P showed that the Spanish version of the TCNS and mTCNS instruments was valid and reliable for use as an instrument for examining diabetic neuropathy.
(14)	Cross-sectional study	625 patients	Independent: Accuracy and Cost-effectiveness of the Diabetic Foot Screen Proforma Dependents: *Diabetic Neuropathy* Detection	Biothesimeter and Diabetic Foot Screen	Measurement of vibration perception threshold (VPT) using biothesimeter and early detection of diabetic foot complications using the Diabetic Foot Screen (DFS)	System 15.0. From ROC analysis and Youden’s index	Vibration perception threshold check (VPT) using DFS was ≥1.5 (sensitivity 62%; specificity 76%), indicating diabetic neuropathy. During the examination, the results were obtained: 74.76% (95% CI: 70.46%-79.06%) of patients experienced diabetic neuropathy. It showed that the use of the DFS DNA biothesimeter can detect diabetic neuropathy early and can be applied to health services
(15)	Cross -sectional study	144 orang	Independent: Michigan Neuropathy Screening Instrument Dependents: Diabetic Peripheral Neuropathy Screening	Michigan Neuropathy Screening Instrument (MNSI), SUDOSCAN, 10-g monofilament test.	Diabetic neuropathy examination using the Michigan Neuropathy Screening Instrument (MNSI), SUDOSCAN, 10 g monofilament test.	Mann–Whitney U test: chi-square test, Spearman.	This combination of instruments can be used for optimal examination of diabetic neuropathy
(16)	Cross-sectional study	10.180 patients	Independent: Machine Learning Michigan Neuropathy Screening Instrument Dependents: Diabetic Sensorimotor Polyneuropathy	*Machine Learning* Michigan Neuropathy Screening Instrument based on Machine Learning	Neuropathy detection using MNSI Machine Learning	performance test: ML Algorithms Correlation: Pearson’s correlation Significant: ANOVA test Correlation Observations and predictions: Cohen’s kappa	Michigan Machine Learning-based Machine Learning can be used to measure diabetic neuropathy. MNSI machine learning ranks in the 10^th^ Class of diabetic neuropathy screening
(17)	cross-sectional study	156 patients	Independent: Ultrasonography (USG) Dependents: Peripheral neuropathy in type 2 diabetes	ultrasonography, Neuropathy Total Score (TNS), Modified Toronto Clinical Neuropathy Screening (MTCNS)	Examination based on ultrasound results and Neuropathy Total Score (TNS), Modified Toronto Clinical Neuropathy Screening (MTCNS)	Shapiro–Wilk test	Ultrasonography (USG) can examine diabetic neuropathy on peripheral nerve ultrasound.
(11)	cross-sectional survey	574 dokter	Independent: Screening and diagnostics Dependents: diabetic polyneuropathy	tripartite questionnaire.	Neuropathy examination with a tripartite questionnaire.	encompassed descriptive	In health care practice in Germany. Patients can use the tripartite questionnaire to screen for diabetic neuropathy
(18)	Cross-sectional study	69 patients	Independent: Frequency Vibration Perception Thresholds Dependents: Diabetic Neuropathy	Customized vibration exciter	Provides vibration to the 1^st^ metatarsal (MTH1) at a frequency of 30 Hz and the heel at a frequency of 200 Hz	Spearman and Pearson, ANOVA	Custom vibration exciters can be used to examine diabetic neuropathy by measuring the vibration perception threshold (VPT) on the metatarsals and heels
(19)	Cross-sectional study	277 patients	Independent: Small and large fiber sensory polyneuropathy Dependents: neuropathy subtypes	128Hz tuning fork, reflex hammer, and pinprick	Vibration perception threshold (VPT) examination uses a 128Hz tuning fork, ankle reflexes were tested with a hammer reflex, and hypoalgesia and hyperalgesia were tested using a pinprick.	Clopper Pearson method	Diabetic neuropathy can be classified into three types, namely, small fiber neuropathy (SFN), large fiber neuropathy (LFN), and mixed fiber neuropathy (MFN).
(20)	Cross-sectional observational study	48 patients	Independent: Conventional Nerve Conduction Studies Dependent: Sensorimotor Polyneuropathy	Biothesiometer, semmes weinstein monofilament (SWMF), nerve conduction studies (NCS), and Michigan Neuropathy Screening Instrument (MNSI)	Neuropathy measurements using a biothesiometer, Semmes Weinstein monofilament SWMF, nerve conduction studies (NCS), and the Michigan Neuropathy Screening Instrument (MNSI)	Independent t-test/Wilcoxon Rank -sum test	Measuring neuropathy using biothesiometry, SRA waves can be done to diagnose neuropathy in a shorter time.
(21)	Cross-sectional study	31 patients	Independent: Conduction nerve interdigital sensory Dependents: Initial diagnosis of Diabetic Neuropathy	Electrode diagnostic	Physical neuropathy examination using diagnostic electrodes was carried out on the sensory nerves consisting of the dorsal nerve, medial plantar nerve, and toes I, II, and III. The filter was set at 2 Hz – 10 kHz, with a speed of 2 sweeps and a sensitivity of 10–20 μV	Mann Whitney U test And the Kolmogorov-Smirnov test	The results showed nine respondents experienced nerve conduction study (NCS) disorders, and 22 subjects were normal. interdigital nerve examination results were abnormal in 17 of 22 patients, whereas nerve conduction studies (NCS) were normal
(4)	Cross-sectional study	104 patients	Independent: Shear wave elastography (SWE) and the Toronto clinical scoring system (TCSS) Dependents: Diabetic peripheral neuropathy	shear wave elastography (SWE) and the Toronto clinical scoring system (TCSS)	SWE examination on peripheral nerve examination with Ultrasonography and Toronto Clinical Scoring System (TCSS)	Evaluated: Mann–Whitney U test Compare: Wilcoxon signed-rank test w Correlation: Spearman correlation	Shear wave elastography (SWE) is an effective tool used to diagnose neuropathy. The combined use of SWE with TCSS is an effective parameter for neuropathy screening
(22)	Cross-sectional study	389 patients	Independent: Diagnosis of neuropathy Dependents: Diabetic neuropathy	The Michigan Neuropathy Screening Instrument (MNSI) and Toronto Clinical Neuropathy Scoring System (TCNS) use the 128 HZ tuning fork tool, biothesimeter, and monofilament thread.	Neuropathy examination used the Michigan (MNSI), Toronto Clinical Neuropathy Scoring System (TCNS), a 128 HZ tuning fork, a biothesimeter, and a monofilament thread.	Spearman’s rank-order correlation	Early neuropathy examination results were obtained using a questionnaire, and more clinical symptoms of neuropathy were brought compared to neuropathy examinations using only a questionnaire
(23)	Cross-sectional study	153 patients	Independent: Clinical Tools for Peripheral Neuropathy Dependents: Diabetic neuropathy	Neurothesiometer, 10 g semmes-weinstein monofilament, Ipswich touch, DPN Check, Neuropathy Disability Score	Assessment of significant nerve fiber function with neurothesiometer, 10 G Semmes-Weinstein monofilament, Ipswich touch, DPN examination, neuropathy disability score (DNS)	Colmogorov -Smirnov test	Slight nerve fiber function examination with negative results of 97%, sensitivity of 89%, and specificity of 73%. In a study using the vibration perception threshold, a pessimistic prediction of 91%, sensitivity of 62%, and specificity of 75% were obtained.
(24)	cross-sectional study.	83 patients	Independent: Turkish version of the Michigan Neuropathy Screening Instrument Dependents: Diabetic peripheral neuropathy	Michigan Neuropathy Screening Instrument Turkish version and Toronto clinical scoring system	Pemeriksaan neuropati menggunakan Michigan *Neuropathy Screening Instrument* version Turki dan Sistem penilaian klinis Toronto	intraclass correlation coefficient, Cronbach’s alpha	The Turkish version of the Michigan Neuropathy Screening Instrument (MNSI) can be used to measure neuropathy symptoms
(25)	Cross sectional study	5088 patients	Independent: Predicting Diabetic Neuropathy Dependents: Artificial Neural Networks and Clinical Parameters	Neurothesimeter	Vibration perception threshold (VPT) measurement using a neurothesimeter	Neural network toolbox on the MATLAB platform	Evaluation of the risk of diabetic neuropathy was carried out using a neurothesimeter and recording the risk factors experienced by the patient. Neurothesimeter examination was categorized into three risks: low at 0-20.99 Volts, medium at 21-30.99 Volts, and high at ≥31 Volts
(26)	Cross sectional study	518 patients	Independent: Artificial intelligence Dependents: Diagnosis of peripheral neuropathy	Artificial intelligence (AI)	Neuropathy examination using Figure cornea identified with AI	Cohen’s κ score	The use of artificial intelligence (AI) to detect neuropathy in people with diabetes by examining the cornea can be done to see neuropathy early.
(27)	Cross sectional study	421 patients	Independent: Vibration perception threshold Dependents: Diabetic polyneuropathy	Neurothesimeter	Vibration perception threshold (VPT) measurement using a neurothesimeter	Mann Whitney	The neurothesimeter can be used to examine diabetic neuropathy by measuring the vibration perception threshold (VPT)
(28)	Cross sectional study	221 patients	Independent: Detection of peripheral neuropathy Dependents: Type 2 diabetes mellitus patient	Michigan Neuropathy Screening Instrument (MNSI) and electrochemical skin conductance (ESC)	Diabetic neuropathy was measured using the Michigan Neuropathy Screening Instrument (MNSI) and electrochemical skin conductance (ESC) on the patient’s hands and feet.	ANOVA test	MNSI and electrochemical skin conductance (ESC) can detect neuropathy in small fiber neuropathy.
(29)	Case control study	60 patients	Independent: Corneal Nerve Plexus Dependents: Diabetic Peripheral Neuropathy	Inspection of early neuropathy diabetes with subbasal nerve plexus (SNP). Inspection done with the method see Rostock Cornea Module (HRT-RCM) and Eye Guidance module (EG) for subbasal nerve plexus (SNP), which indicates neuropathy diabetes.	Rostock Cornea Module (HRT-RCM) and EyeGuidance module (EG)	Mann–Whitney test	Diabetes examination is divided into three categories: corneal nerve fiber length (CNFL; mm/mm2), corneal nerve fiber density (CNFD; no./mm2), corneal nerve branch density (CNBD; no./mm2). Based on this, it showed that in assessing diabetic neuropathy using SNP at an early stage, there were no differences in neuropathy in diabetes mellitus patients.
(30)	Case control study	341 patients	Independent: Neuropathy screening tool Dependents: Diabetic sensorimotor polyneuropathy	Toronto Clinical Neuropathy Score (TCNS)	Neuropathy examination with the Toronto Clinical Neuropathy Score (TCNS)	ANOVA tests	Patient assessment using the Toronto Clinical Neuropathy Score (TCNS). Screening by examining the hand cold detection threshold (CDT), hand warm detection threshold (WDT), foot CDT, and foot WDT. Early detection neuropathy more accurate by clinical symptoms.

**Table 3:** Neuropathy detection based on review.

Type Instrument	Author	Number	Methods examination
Vibration perception threshold or Biothesimeter	(18), (23), (25), (27), (14), (20), (22)	7 articles	VPT is a vibration activated under controlled pressure, there is pressure monitoring and the elasticity of the vibration is electrically controlled in both directions with five indicator lights. VPT adopted this new technology biothesiometer to assess VPT trends in subjects without sensorimotor distal symmetric polyneuropathy and identify age-specific normality thresholds. The voltage is given starting from 0.5 volts. Patients are considered to have neuropathy if they do not feel a voltage of ≥ 25 mV
Michigan Neuropathy Screening Instrument	(33), (15), (20), (22), (24), (28)	6	Interview with questions on the questionnaire with 15 questions about sensory perception. The result if the patient neuropathy, will answer ≥ 7 questions.
Toronto clinical neuropathy score (TCNS)	(30), (13), (17), (4), (22), (24), (30)	7	TCNS is to know level severity with check symptoms and sensitivity in the patient’s feet. The tool use Questionnaire Toronto, reflex examination and sensory test score. Questionnaire consist 6 symptoms: Pain, Numbness, tingling, weakness, ataxia, upper limb symptom. Ask patient about present (score 1) or absence (score 0) of symptom. After that reflex examination to knee and ankle reflex result Absence: score 2, Reduce: score 1, Normal: score 0). Sensory Test Score include pinprick, temp, light touch, vibration, position. The result sensory Abnormal (score 1), Normal (score 0). Conclusion TCNS: No neuropathy 0-5 points, Mild neuropathy 6-8 points, Moderate neuropathy 9-11 points, Severe neuropathy 12+ points.
tripartite questionnaire.	(11)	1	This questionnaire is divided into 3 parts: the first part contains participant data, the second part contains the neuropathy examination procedures, and the third part contains questions regarding the examination of pain, sensitivity, and temperature sensation.
128Hz tuning fork, reflex hammer, and pinprick	(19)	1	Inspection done on the instep, Inspect Vibration with 128 Hz tuning fork, sensation temperature cold with tuning fork, sensation puncture needle with monofilament test, Achilles tendon reflex with use patellar hammer.
Electrode diagnostic	(21)	1	The examination uses an electrode with a current of <25 mA with a distance between the electrode and the stimulus depending on the size of the foot of 8-10 cm. Nerve action potential (NAP) was considered absent if it was not recorded at >20 mA indicating neuropathy
shear wave elastography (SWE	(4)	1	Shear wave elastography (SWE) detects neuropathy by looking at images of the nerves in the tibial area which indicates neuropathy if the results show nerve stiffness in the tibial area
10 g semmes-weinstein monofilament	(15), (20), (22), (23)	4	Push monofilament 10 gr thread on point- point specifically on the feet. Ask the patient to close his eyes, The nurse explains that they will check the feet in several places, say “yes” if the patient feels it or if the patient does not feel it. Hold the monofilament to the skin perpendicularly, bending it, and then holding it back perpendicularly for about 1.5 seconds. Examine the plantar toes 1, 3, 5, metatarsal heads of toes 1, 3, 5, medial and lateral arches, heel and dorsum of the foot
Artificial intelligence (AI)	(26)	1	Neuropathy examination with AI using the Heidelberg Retina Tomograph III using the Rostock Corneal Module (RCM) to view the cornea in diabetes patients
electrochemical skin conductance (ESC)	(28)	1	Electrochemical skin conductance (ESC) detects neuropathy using electrodes connected to a computer. Electrodes are attached to the feet and hands, and then connected to a computer. This tool measures the response of skin conductance to electric current given through an electrode and then connects the results to a computer
Rostock Cornea Module (HRT-RCM) and EyeGuidance module (EG)	(29)	1	The examination includes patient demographic data, subsequent examination using an ophthalmological slit lamp and ophthalmoscopy to determine retinopathy, then examination using the Heidelberg Retina Tomograph II equipped with the Rostock Cornea Module (HRT-RCM) and Eye Guidance (EG) to result corneal confocal microscopy (CCM) in quantifying nerve fiber abnormalities in diabetic neuropathy
SUDOSCAN	(15)	1	SUDOSCAN is a test that provides an accurate evaluation of sweat function. The test focuses on small nerve fibers within the peripheral nervous system innervating the sweat glands. The device consists of a computer and 4 electrodes on which patients place their hands and bare feet. In less than 3 minutes, SUDOSCAN offers a stimulation of the sweat glands that assess nerve C fibers.
Neuropathy Total Score (TNS)	(17)	1	In the TNS examination, there are 8 parts: sensory and motor symptoms, pricking sensation, vibration sensation, strength examination, deep tendon reflexes, sural sensory amplitude, and tibial motor amplitude. TNS assessment score 1-4 with a total score of 32. Examination results are categorized into 4: level 0: 0-1, level 1: 2-8, level 2: 9-16, level 3: 17-24, level 4: 25- 32.
Neuropathy Disability Score	(23)	1	NDS examination of small nerve fibers uses pricking sensation and temperature sensation. This examination is considered positive neuropathy if there is damage to one of the 2 examination points in the lower extremity
DPN Check	(23)	1	DPN-Check is used for automatic sural nerve conduction examination. examination results show neuropathy if the amplitude is 4 or the conduction velocity is 40m/s in 1 of the 2 lower extremities
Ipswich touch	(23)	1	Ipswich touch examination of the feet with pressure using the index finger on the 1st, 3rd, and 5th toes of the lower extremities. The results show neuropathy if you don’t feel two touches

## Discussion

The final results of this review were 20 pieces of literature that discussed early detection of neuropathy in diabetes patients. Researchers used diabetic neuropathy instruments to carry out early detection of neuropathy. Of the 20 literatures, there were 20 literatures that showed good results in diabetic neuropathy examination. The results of JBI’s critical appraisal risk of bias, show that the journals included in this research meet the critical appraisal requirements with an assessment reaching ≥50%. However, in question 3, the assessment was <50%, 3 studies did not include exclusions for samples included in the study, and 3 articles excluded samples because the sample data was empty.

Diabetes Mellitus is a very important health problem in society, the incidence and number of cases of Diabetes Mellitus sufferers has always increased over the past few years (34, 35). Diabetes Mellitus (DM) or diabetes is a heterogeneous group of disorders with typical signs of increased blood glucose levels or hyperglycemia (36). Diabetic patients experience blood glucose resistance for a long time resulting in neuropathy complications. Prolonged high blood glucose levels will result in damage to the blood vessels walls (37). In this condition, the patient’s body cannot use the glucose in the blood to convert it into energy due to the accumulation of glucose in the blood (38). Neuropathy may cause damage to sensory, motor, and autonomic nerves. The clinical symptoms felt by diabetes patients are based on the damaged nerves, for example motor neuropathy (deformity), sensory neuropathy (decreased sensitivity), and autonomic neuropathy (callus). To confirm the diagnosis of neuropathy, health workers can carry out early detection of neuropathy (3).

Delay in early diagnosis of neuropathy may cause the severity of neuropathy and development of diabetic ulcers. The length of time a person living with diabetes can provide an idea of the course of the disease and also the person’s severity (37). Examination results showed severe neuropathy identify the risk of diabetic ulcers (39). Patients suffering from neuropathy will experience decrease in quality of life because they experience symptoms of neuropathy such as pain, deformity and callus (3).

Diabetic neuropathy examination can be carried out using instruments that are available in health services. However, most neuropathy instruments can only detect after the patient has symptoms of neuropathy. For instance, monofilament instruments can detect neuropathy that has decreased sensitivity in the feet (40), the MNSI questionnaire can identify neuropathy based on signs of neuropathy symptoms felt by diabetics (41).

Vibration perception threshold (VPT): there are 7 studies using different instruments in the vibration perception threshold measurement method, namely biotesimeter (14, 20), neurotesimeter (23) (25) (27), (18) vibratip, 128 Hz tuning fork (19). During the VPT examination, researchers provided vibrations at certain points to detect vibration sensations in the feet of diabetic patients. The vibration range given during the VPT examination was from 1-50V with the result categories being mild neuropathy, moderate neuropathy and severe neuropathy.

Michigan neuropathy screening instrument (MNSI): there are 7 studies using the MNSI in early screening for diabetic neuropathy. The MNSI questionnaire consists of 11 questions regarding signs and symptoms of neuropathy in diabetes mellitus patients. Researchers have developed the MNSI questionnaire, there is a research that has developed the MNSI in the form of machine learning, the MNSI is available in various versions such as the Turkish version of the MNSI (33).

Toronto clinical neuropathy score (TCNS): there are 7 studies that use TCNS in the examination of diabetic neuropathy. Researchers translated the TCNS into Spanish (13). In another study, TCNS was modified into the modified Toronto clinical neuropathy score (m-TCNS) instrument (m-TCNS) (17).

Other examinations: Diabetic neuropathy examination, apart from using the above instruments, can also use ultrasonography (USG), tripartite questionnaire, electrode diagnostic, shear wave elastography artificial neural network and artificial intelligence, cornea module. The results based on neuropathy examination using this instrument were normal and neuropathic. However, this instrument is rarely used in health services.

The research used a combination of early detection methods for neuropathy: other research used a combination of the early detection instruments mentioned above and combined using other instruments such as monofilament, sudoscan, electrochemical skin conductance, Ipswich touch, neuropathy disability score and Hammer reflex. Instruments for early detection of neuropathy in each study can also be seen in detail in **Table 2**.

Over time, many researchers have developed instruments for early detection of diabetic neuropathy. The development of this instrument can make it easier for health workers to detect neuropathy early and determine appropriate treatment so as to prevent the occurrence of diabetic ulcers. For example, researchers translated the Turkish version of the MNSI questionnaire so that it can be used by Turkish health workers in detecting neuropathy (24). Based on existing research, examination of diabetic neuropathy can use artificial intelligence instruments by looking at images of the cornea in diabetes mellitus patients (26).

The results of the diabetic neuropathy examination are stated to be in accordance with the instrument used. There is an instrument that describes the results of neuropathy with 3 classifications, namely mild neuropathy, moderate neuropathy and severe neuropathy (41). Furthermore, there are instruments that identify neuropathy with normal results and neuropathy. Health workers can combine instruments for early detection of neuropathy so that examination results are more accurate (42).

A study using the Toronto Clinical Neuropathy Score (TCNS), hand cold Detection Threshold (CDT), Hand Warm Detection Threshold (WDT), Foot CDT, and Foot WDT instruments in diagnosing neuropathy did not show accurate results. Early diagnosis of neuropathy is more accurate through the patient’s clinical symptoms. Based on this, the doctor confirms the diagnosis of neuropathy after the patient feels clinical symptoms (30).

Neuropathy examination in diabetes patients using an early neuropathy detection tool. Various neuropathy screening tools are available with different assessment methods. The neuropathy questionnaire instrument detects neuropathy through clinical symptoms. Questionnaire questions cover patient symptoms such as pain, deformity, and decreased sensitivity. Physical examination of neuropathy using a monofilament instrument and a tuning fork.

Based on the articles included in this study, it discusses the sensitivity and specificity of neuropathy instruments. The sensitivity and specificity of the instrument show the accuracy of the instrument in diagnosing neuropathy. Validity measurements in articles use different methods including validity tests using Cronbach’s alpha and ROC/AUC assessments. We found vibration perception threshold examination (biothesimener/neurothesimeter/vibratip) is the most frequently used physical examination instrument for neuropathy detection with a sensitivity value of 62%; and specificity of 76%). Vibrations of 1-50V are given to the patient’s feet at several examination points, indicating neuropathy if they feel vibrations ≥25V and no neuropathy if they feel <25V. The vibration perception threshold instrument has become the gold standard for detecting neuropathy and ulcer risk (14).

Apart from physical examination instruments using the vibration perception threshold, the MNSI questionnaire is also the most frequently used in the early detection of neuropathy. This questionnaire consists of 15 questions regarding neuropathy symptoms with the results identifying neuropathy into 3 categories, namely low, moderate, and severe (15). Several studies combine the MNSI questionnaire with physical examination tools such as monofilaments and tuning forks. The MNSI questionnaire has been adapted and translated into various languages including Indonesian, Arabic, and Thai.

Meanwhile, neuropathy instruments such as the Toronto Clinical Neuropathy Score (TCNS, ultrasonography (USG), tripartite questionnaire, diagnostic electrodes, artificial neural network shear wave elastography, and artificial intelligence, cornea modules are still rarely used in diabetic neuropathy examination. Research also combines instruments of Early detection to get accurate results.

## Conclusion

This review reports on the importance of early detection of neuropathy for diagnosing neuropathy and determining appropriate management. Neuropathy patients who receive appropriate treatment can prevent the occurrence of diabetic ulcers. The most frequently used neuropathy instruments are the vibration perception threshold (VPT) and questionnaire Michigan Neuropathy Screening Instrument (MNSI). Health workers can combine neuropathy instruments to accurately diagnose neuropathy.

## Author contributions

OP: Conceptualization, Investigation, Methodology, Resources, Software, Visualization, Writing – original draft, Writing – review & editing. N: Supervision, Validation, Writing – original draft, Writing – review & editing, Methodology. MP: Supervision, Validation, Writing – original draft, Writing – review & editing.
